# Mesenchymal stem cells from different sources show distinct therapeutic effects in hyperoxia‐induced bronchopulmonary dysplasia in rats

**DOI:** 10.1111/jcmm.16817

**Published:** 2021-07-29

**Authors:** Yingjun Xie, Fei Chen, Lei Jia, Rui Chen, Victor Wei Zhang, Xinqi Zhong, Ding Wang

**Affiliations:** ^1^ Department of Obstetrics and Gynecology Key Laboratory for Major Obstetric Diseases of Guangdong Province The Third Affiliated Hospital of Guangzhou Medical University Guangzhou China; ^2^ Key Laboratory of Reproduction and Genetics of Guangdong Higher Education Institutes The Third Affiliated Hospital of Guangzhou Medical University Guangzhou China; ^3^ Reproductive Medicine Research Center Sixth Affiliated Hospital of Sun Yat‑Sen University Guangzhou China; ^4^ AmCare Genomics Lab Guangzhou China; ^5^ Department of Pediatrics The Third Affiliated Hospital of Guangzhou Medical University Guangzhou China

**Keywords:** bronchopulmonary dysplasia, different source, inflammation, mesenchymal stem cells, neovascularization

## Abstract

Mesenchymal stem cells (MSCs) have been shown as an effective medicinal means to treat bronchopulmonary dysplasia (BPD). The widely used MSCs were from Wharton's jelly of umbilical cord (UC‐MSCs) and bone marrow (BM‐MSCs). Amniotic fluid MSCs (AF‐MSCs) may be produced before an individual is born to treat foetal diseases by autoplastic transplantation. We evaluated intratracheal (IT) MSCs as an approach to treat an hyperoxia‐induced BPD animal model and compared the therapeutic effects between AF‐, UC‐ and BM‐MSCs. A BPD animal model was generated by exposing newborn rats to 95% O_2_. The continued stress lasted 21 days, and the treatment of IT MSCs was conducted for 4 days. The therapeutic effects were analysed, including lung histology, level of inflammatory cytokines, cell death ratio and state of angiogenesis, by sacrificing the experimental animal at day 21. The lasting hyperoxia stress induced BPD similar to the biological phenotype. The treatment of IT MSCs was safe without deaths and normal organ histopathology. Specifically, the treatment was effective by inhibiting the alveolar dilatation, reducing inflammatory cytokines, inducing angiogenesis and lowering the cell death ratio. AF‐MSCs had better therapeutic effects compared with UC‐MSCs in relieving the pulmonary alveoli histological changes and promoting neovascularization, and UC‐MSCs had the best immunosuppressive effect in plasma and lung lysis compared with AF‐MSCs and BM‐MSCs. This study demonstrated the therapeutic effects of AF‐, UC‐ and BM‐MSCs in BPD model. Superior treatment effect was provided by antenatal MSCs compared to BM‐MSC in a statistical comparison.

## 1 INTRODUCTION

Bronchopulmonary dysplasia (BPD) is a chronic lung disease major in preterm infants characterized by arrest of alveolation, fibroblast activation and inflammation. Some patients have fibrosis of the lungs caused by prolonged mechanical ventilation and oxygen exposure.^1^ The incidence rate of BPD was up over 30% in premature infant in the United States^2^ and Europe,^3^ regardless of race. For antenatal, the risk factors of BPD included maternal smoking and intrauterine growth restriction (IUGR). For postnatal cases, the risk factors were hyperoxia and mechanical ventilation.^2^ BPD increases mortality in neonates, and it is the leading cause of chronic lung disease in children.^4^ Adult survivors of BPD also presented with pulmonary impairment‐associated diseases^5^ and also had long‐term cardiopulmonary morbidities.^2^ The clinical treatments for BPD were dependent on their manifestation. Old BPD, characterized by fibrosis and inflammation, were cured by the discovery of effective biomaterial and ventilation devices. However, new BPD, marked by tissue simplification and arrest of alveolarization, still affect premature infants.^6^, ^7^ Cytotherapy, namely, mesenchymal stem cell (MSC)‐based therapy, provides a new possibility to effectively treat BPD.

MSCs are cultured primary cells, which are defined with specific bio‐characteristics^8^ in vitro and bio‐functions in vivo, namely, tissue protection, inflammation regulation and promotion of angiogenesis.^9^ Likewise, MSC‐derived conditioned media conferred therapeutic benefit for alveolarization, pulmonary artery remodelling and angiogenesis.^10^ These cells may be derived at the prenatal stage,^11^, ^12^ such as amniotic fluid MSCs (AF‐MSCs) and umbilical cord MSCs (UC‐MSCs), and postpartum,^13^, ^14^, ^15^ such as bone marrow MSCs (BM‐MSCs). Although MSCs are derived from different sources and share similar bio‐characteristics and bio‐functions, their therapeutic effects still remain unknown. MSCs were shown to be low immunogenicity bio‐products for allogeneic transplantation schemes and have been widely used in clinical trials. Recently, the therapeutic effects of MSCs were demonstrated by an animal model study and clinical trial in several acute and chronic injury disease examples. During the epidemic period of the coronavirus disease 2019 (COVID‐19), MSCs^16^ and MSC‐derived extracellular vesicles (EVs)^17^ were reported as an effective therapeutic scheme in the treatment of infected patients. For BPD, the therapeutic effects of MSCs were shown in an animal disease model^10^ and clinical trial.^4^ MSC BPD treatment led to relieve of lung injury, which improved the histology, reduced inflammation and increased angiogenesis.

Treatment with MSCs in preclinical hyperoxic models of BPD in rodents resulted in statistically significant improvement in lung injury.^10^ MSCs may be infused for treatment by intravenous,^2^ intraperitoneal (IP) or intratracheal (IT) administration. The latter is the first choice for MSCs for the treatment of BPD by being safe and effective as showed in animal studies^18^ and clinical treatment,^19^ even used in some clinical phase one study.^20^, ^21^ However, the therapeutic effect of MSCs from different sources has not been investigated.

In this study, we generated a hyperoxia‐induced neonatal lung injury model to mimic BPD. The modelling rat got the physiological manifestation correspond with BPD pathology, such as simplification of lung histology, inflammation and cell death increase and decrease of neovascularization. Then, we IT administration of AF‐, UC‐ and BM‐MSCs and tested their therapeutic effect in our BPD model. We showed that the hyperoxia‐induced pathological changes were relieved by all treatments; however, physiological and biochemical difference between the groups were observed. Collectively, we demonstrated the safety and effectiveness in improving hyperoxia‐induced BPD‐like pathological changes by IT MSCs, and MSCs from prenatal (AF‐ and UC‐MSCs) cells and illustrated several advantages compared with postpartum (BM‐MSCs) therapy.

## 2 MATERIALS AND METHODS

### 2.1 BPD model and MSC cytotherapy

**FIGURE 1:**
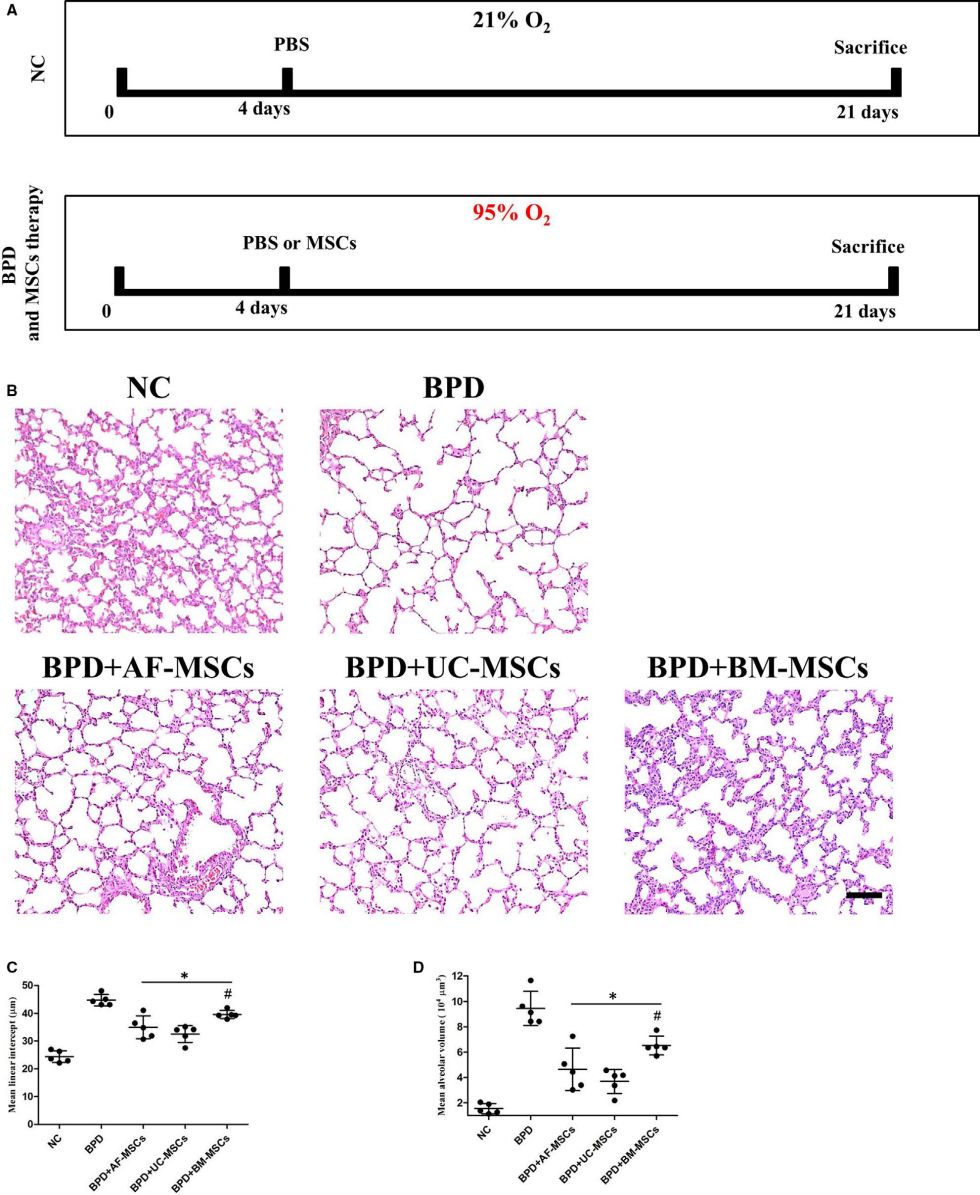
Intratracheal administration of MSCs relieves hyperoxia‐induced lung histological changes in neonatal rats. Schematic representation of the time course of BPD modelling and MSC therapy (A). Non‐specific control (NC) was set up in normoxia (21% O2), bronchopulmonary dysplasia (BPD) was modelling by hyperoxia atmosphere (95% O2). 50 μl PBS or 50 μl PBS with 5 × 10^5^ MSCs from different sources was intratracheal administration on day 4, and all animals were sacrificed on day 21 for further study. The histology of the lung in different groups was identified using HE staining (B). IT for MSCs ameliorated hyperoxia‐induced alveolar expansion, according to the mean linear intercept (C) and mean alveolar volume (D). *n* = 5; *, *p* < 0.05 compared with the BPD group, #, *p* < 0.05 compared with the BPD+UC‐MSC group. Scale bar=100 μm

The Sprague Dawley (SD) rats (Guangdong Medical Laboratory Animal Center, Guangzhou, China) on the first day after birth were used to generate BPD rats by exposing them to hyperoxic conditions (95% O2), in which animals were raised in a Hypoxia/Hyperoxia incubator (PH‐A1, Puhe bio, Wuxi, China). The BPD rats were fed by lactation by one rat for one day and changed to other lactating rats kept in normoxia (21% O2). In addition, non‐specific control (NC) neonatal rats were raised in normoxia (21% O2). Neonatal rats transferred to hyperoxic conditions regarded as day one, cytotherapy using MSCs was performed on day 4 via a different MSC intratracheal injection (*n* = 5 for each group, 5 × 10^5^ cells in 50 μl of PBS per animal). As a control, NC (*n* = 5) and BPD rats (*n* = 5) were given 50 μl of PBS without cells. All of the experimental animals were sacrificed on day 21 for further study (Figure 1A). This study was approved by the Academic Committee of the Third Affiliated Hospital of Guangzhou Medical University.

### 2.2 Cell culture

The human amniotic fluid MSCs (AF‐MSCs) and umbilical cord MSCs (UC‐MSCs) were isolated and characterized as previously described.^11^ Human bone marrow MSCs were a gift from the Center for Stem Cell Biology and Tissue Engineering of Sun Yat‐sen University. The derivation and characterization of these cells was previously reported.^22^ The maintenance and expansion of MSCs was performed using Yu's protocols.^22^

### 2.3 Histology and tissue staining

After animals were sacrificed, several organ histology was completed on liver, kidney, heart, spleen, brain, lymph, thymus and lung. Organs were fixed with 4% paraformaldehyde, dehydrated and embedded in paraffin. The paraffin sections were stained using haematoxylin‐eosin (HE) (Cat# G1121, Solarbio, Beijing, China). For hyperoxia‐affected lungs only, the paraffin sections of lungs were stained using the TUNEL (TdT‐mediated dUTP Nick‐End Labelling) staining kit according to the manufacturer's instructions to identify apoptotic cells (Cat# G3250, Promega (Beijing) Biotech Co., Beijing, China). CD34‐positive cells were identified using the metal‐enhanced DAB substrate kit (Cat# DA1015, Solarbio) after CD34 antibody staining (Cat# sc19621, Santa Cruz Biotechnology, Shanghai, China, dilution 1:50). The pictures were collected using a Zeiss microscope (Axio Imager. A2, Carl Zeiss, Gottingen, Germany).

### 2.4 Biochemical assay

The concentration of secreted cytokines in plasma and lung lysates were determined by ELISA (enzyme‐linked immunosorbent assay). Plasma was separated by centrifuging whole blood without anticoagulation which was held at room temperature for 2 h after drawing. For tissue lysates, 0.1 g of fresh lung tissue was homogenized in 1 ml of lysis buffer using a tissue grinder (Tissuelyser 24, Shanghai Jingxin Industrial Development CO., LTD, Shanghai, China). The VEGFA kit was purchased from the Cloud‐Clone Corp. (Cat# SEA143Ra, Katy, TX, USA). Other kits were purchased from Cusabio technology LLC (Wuhan, Hubei, China), namely, IL1B (Cat# CSB‐E08055r), IL6 (Cat# CSB‐E04640r), ET1 (Cat# CSB‐E06979r) and TNF‐α (Cat# CSB‐E11987r).

### 2.5 Western blot

Western blotting was carried out as previously described.^23^ Protein of each sample was quantified by BCA protein concentration assay kit (Cat# PC0020, Solarbio, Beijing, China). The loading quantity was 30 μg of each sample, and proteins were distinguished by SDS‐PAGE (sodium dodecyl sulphate‐polyacrylamide gel electrophoresis) and transferred to PVDF (polyvinylidene fluoride) membrane for blotting. For CD34, the antibody for Western blot was the same as for immunohistochemistry, and dilution was 1:1000. The other antibodies were purchased from Abcam (Shanghai, China), namely, CD31 (Cat# ab28364, 1:500), CDH5 (Cat# ab33168, 1:1000) and VEGFA (Cat# ab1316, 1:500).

### 2.6 Statistical analysis

Quantitative results for Western blot was expressed as the mean ± standard deviation. The other data were demonstrated in scatter plot. The data of multiple groups were statistically analysed by ANOVA. Two‐tailed Student's *t* test was applied to compare the differences between the two groups. A value of *p* < 0.05 was considered to indicate a statistically significant difference.

## 3 RESULTS

### 3.1 MSCs rescued hyperoxia‐induced BPD associated with pathological changes in neonatal rat lung

A BPD model was established on neonatal Sprague Dawley rats by exposing them to hyperoxia (95% O_2_). In addition, non‐specific control (NC) neonatal SD rats were raised in normoxia (21% O_2_). Cytotherapy using MSCs was performed on day 4, which included IT administration of 50 μl PBS with 5 × 10^5^ AF‐MSCs, UC‐MSCs or BM‐MSCs. As a control, NC and BPD rats were given 50 μl of PBS without cells. Furthermore, biological analysis was performed on day 21 by sacrificing five experimental animals from each group (*n* = 5) (Figure 1A). During the experimental treatments, no deaths of infant rats were observed. Morphological changes were observed by HE staining. Hyperoxia did not induce significant pathological changes in main organs, including liver, kidney, heart, spleen, brain, lymph and thymus and so did MSC cytotherapy (Figure S1). For lung histological analysis, hyperoxia induced larger and fewer alveoli in the BPD group compared with the NC normoxia group. Cytotherapy, using MSCs, ameliorated the lung pathological changes (Figure 1B). The mean linear intercept (Figure 1C) and mean alveolar volume (Figure 1D) were used to perform statistical analysis of alveoli. All MSC therapies reduced the hyperoxia‐induced mean linear intercept and mean alveolar volume increased. The UC‐MSC group showed a significant reduction of the histological indexes of alveoli compared with BM‐MSC groups.

### 3.2 MSCs ameliorated hyperoxia‐induced secreted cytokine changes in circulatory system and lung tissue

**FIGURE 2:**
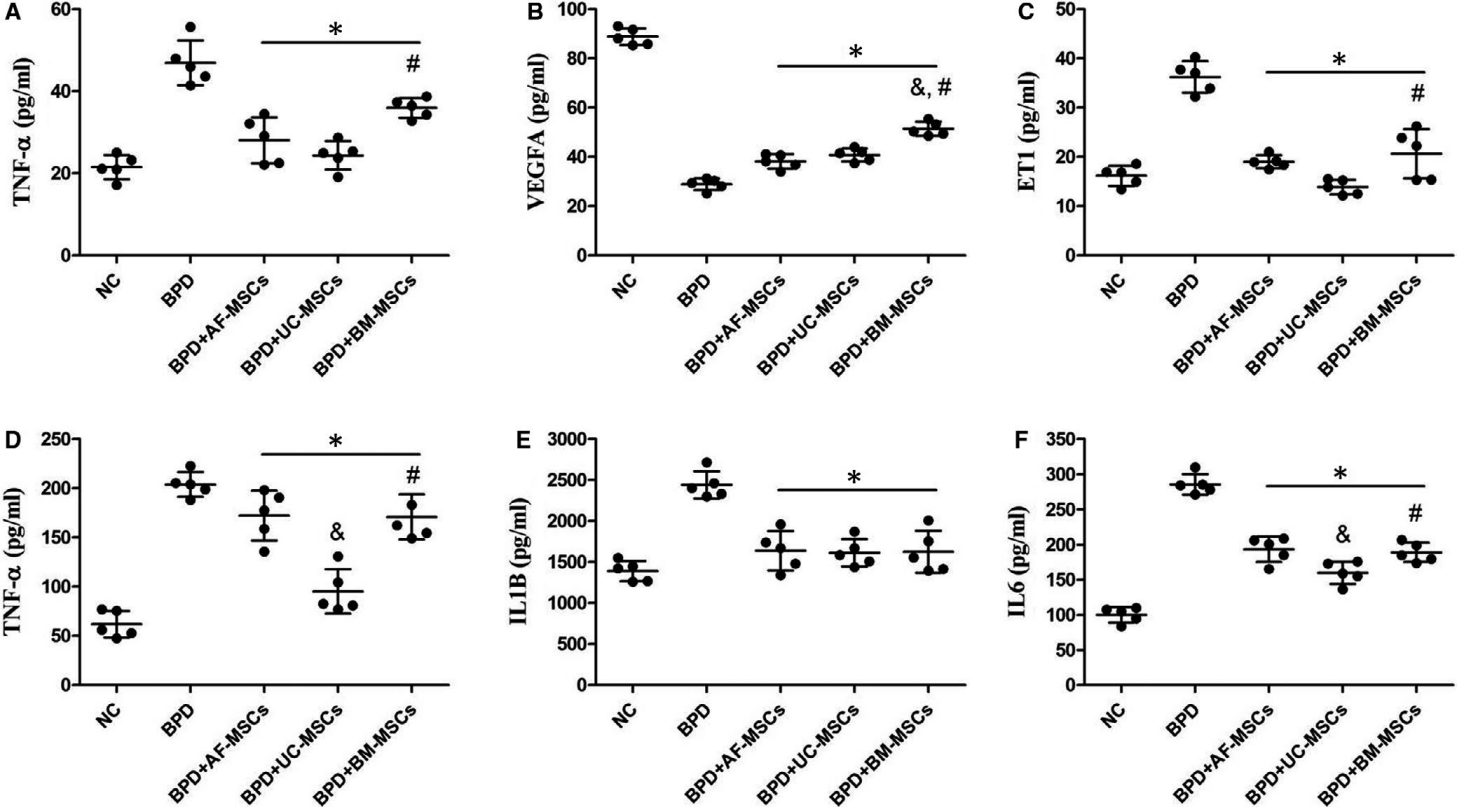
Cytotherapy of MSCs ameliorate hyperoxia‐induced inflammation and angiogenesis‐associated cytokine level changes in plasma and lung tissues in BPD rats. The plasma levels of proinflammatory and angiogenesis cytokines were measured including TNF‐α (A), VEGFA (B) and ET1 (C). Hyperoxia induced increase of TNF‐α and ET1 and decrease of VEGFA, while cytotherapy of MSCs ameliorated these changes. Some other proinflammatory cytokines were measured in lung tissue lysis, including TNF‐α (D), IL1B (E) and IL6 (F). Cytotherapy of MSCs also ameliorated hyperoxia‐induced proinflammatory cytokine increase. *n* = 5; *, *p* < 0.05 compared with the BPD group; &, *p* < 0.05 compared with the BPD+AF‐MSC group; #, *p* < 0.05 compared with the BPD+UC‐MSC group

The secreted cytokines from rats were assayed using ELISAs. For the circulatory system, three plasma cytokines were assayed, including proinflammatory factor TNF‐α (Figure 2A), angiogenesis‐associated factor VEGFA and ET1 (Figure 2B and C). After IT administration of MSCs, the hyperoxia‐induced group had an increase in TNF‐α and ET1 levels and a decrease in VEGFA level. Moreover, there were significant increases in TNF‐α and ET1 levels in the BPD+BM‐MSC group compared with BPD+UC‐MSCs and a significant increase in VEGFA level in the BPD+BM‐MSC group compared to the BPD+AF‐MSC and BPD+UC‐MSC groups. For lung tissue, three proinflammatory factors were assayed, including TNF‐α, IL1B and IL6 (Figure 2D, E and F). There was a significant alleviation of the BPD phenotype by the three MSC administrations. Similarly, the same trends were seen in other MSC group comparisons as measure by TNF‐α and IL6.

### 3.3 MVD and cell death in lung tissue were improved by MSC cytotherapy

**FIGURE 3:**
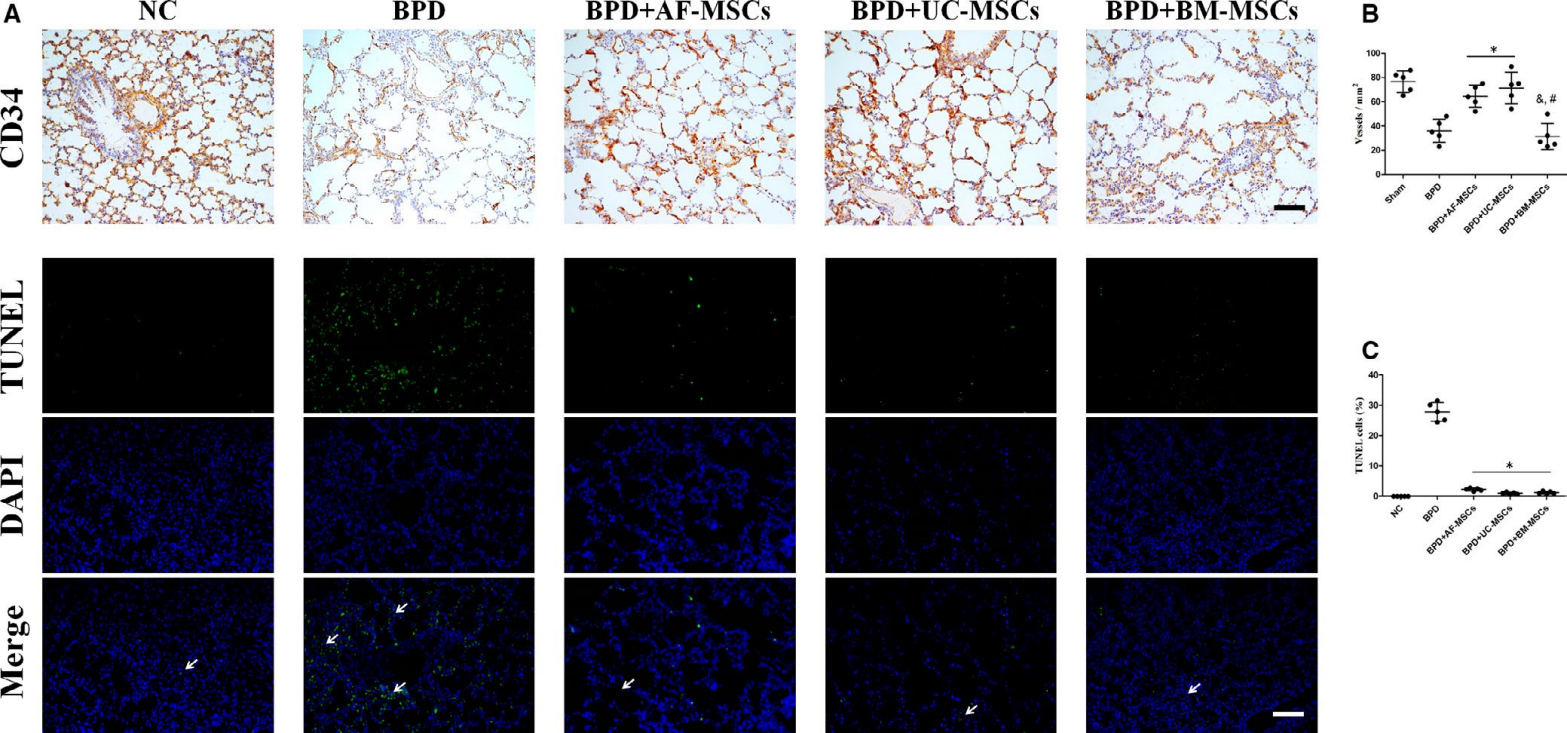
Administration of MSCs improves hyperoxia‐induced lung neovascularization decrease and cell death in BPD rats. Vascularization of lung was identified by CD34 immunohistochemistry (A). Cell death of lung was identified by TUNEL staining (A, the green range indicated by the arrow). Statistical analysis of microvessel density (MVD) change and cell death in BPD and BPD with MSC cytotherapy was indicated by vessels per mm^2^ (B) and percentage of TUNEL‐positive cells (C). *n* = 5; *, *p* < 0.05 compared with the BPD group; &, *p* < 0.05 compared with the BPD+AF‐MSC group; #, *p* < 0.05 compared with the BPD+UC‐MSC group. Scale bar=100 μm

Immunohistochemistry with the CD34 antibody was used to discriminate vessels, and TUNEL staining labelled the apoptotic cells in lung tissue (Figure 3A). In the NC group, most of alveoli were covered by CD34‐positive vessels and there a large CD34‐positive vessel loss in the BPD group, which were partly reversed by MSC cytotherapy. Statistical analysis of microvessel density showed a significant increase in MDV in BPD+AF‐MSC and BPD+UC‐MSC groups compared with the BPD group. No MDV significant change was seen in the BPD and BPD+AF‐MSC group comparison. However, a significant MDV decrease was observed in the BPD+BM‐MSC group compare with BPD+AF‐MSC and BPD+UC‐MSC groups (Figure 3B). For cell death statistical analysis, hyperoxia‐induced apoptosis was attenuated by all MSC cytotherapies, and there was no significant difference between MSC groups (Figure 3C).

### 3.4 Hyperoxia‐induced angiogenesis and epithelialization‐associated protein degradation in lung was ameliorated by MSC cytotherapies

**FIGURE 4:**
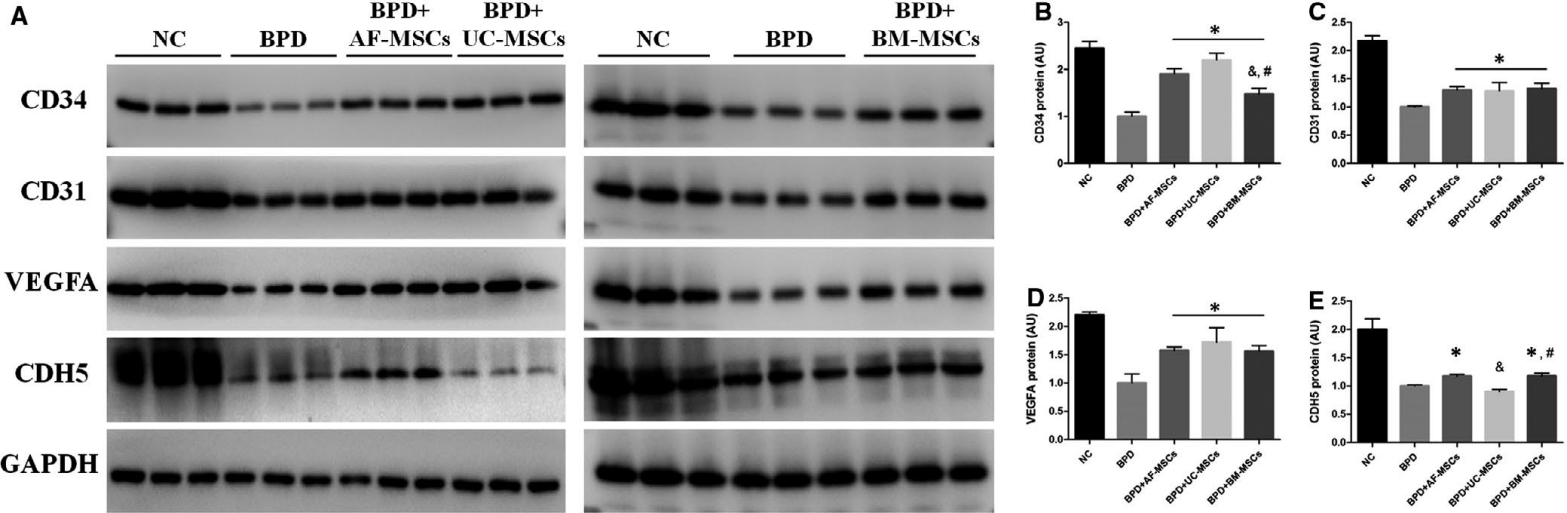
Administration of MSCs inhibits hyperoxia‐induced lung angiogenesis and epithelialization‐associated protein degradation in BPD rats. The lung tissue protein expressions of angiogenesis were assayed by Western blotting including NC, BPD modelling and BPD treated with MSCs (A). Statistical analysis of protein expressions was based on the grey level of Western blotting band, including CD34 (B), CD31 (C), VEGFA (D) and CDH5 (E). *n* = 3; *, *p* < 0.05 compared with the BPD group; &, *p* < 0.05 compared with the BPD+AF‐MSC group; #, *p* < 0.05

The protein level in lung tissue was determined by Western blotting for angiogenesis (CD34, CD31 and VEGFA) and epithelialization (CDH5) markers (Figure 4A). There was significant decrease in all markers in the BPD group compared with the NC group. For the angiogenesis‐associated markers, a similar trend was present, as there were significant increases in the CD34, CD31 and VEGFA levels in MSC cytotherapy groups compared with the BPD group (Figure 4B, C and D), while there was a significant decrease in CD34 levels in the BPD+BM‐MSC group compared with BPD+AF‐MSC and BPD+UC‐MSC groups. CDH5 is regarded as an epithelial marker, which was significantly decreased by the hyperoxia‐induced BPD, and the decrease was inhibited by AF‐MSC and BM‐MSC treatments but not by UC‐MSCs. Moreover, there was a significant CDH5 level decrease in the BPD+UC‐MSC treatment group compared to the BPD+AF‐MSC and BPD+BM‐MSC groups (Figure 4E).

## 4 DISCUSSION

BPD pathology may be mimicked by hyperoxia‐induced lung injury in neonatal rats. Hyperoxia‐induced oxidative stress is one of the most important postnatal risk factors for BPD.^2^ The intrauterine growth atmospheric oxygen is 4% O_2_, and the hyperoxia environment for newly born neonates is the exposure to 21% O_2_.^24^ The reactive oxygen species^1^ are generated during this environmental oxygen change. Hyperoxia^25^ and ROS^26^ are both the inducer of cell death and tissue damage. Premature infants possess a lower level of antioxidant defences and higher susceptibility to infection and inflammation,^27^ which exacerbate lung injury. Thus, hyperoxia‐induced lung injury is the most commonly used animal model in study BPD’s MSC cytotherapy.^10^ We generated a BPD model by exposing neonatal SD rats to 95% O_2_. No animals died during the hyperoxia exposure including the animal model groups and MSC treatment groups. The lung pathological changes of the BPD model were consistent with clinical manifestation, including alveolar expansion, higher inflammatory cytokines, cell death induction and angiogenesis loss.

Because of the lung's unique accessibility via the airways, we used the intratracheal route of delivery. A strength of the intratracheal approach is that it mimics the clinical setting and could theoretically be used (if safe and effective) to treat premature infants who are likely to develop BPD, concomitantly with routine surfactant administration. It is safe to treat BPD model rats by IT MSCs, which has been reported to be safe without causal of serious adverse events after either infusion or instillation of MSCs^28^ for the treatment of respiratory diseases by meta‐analysis of clinical trials.^29^ IV leading the lethal risk of thromboembolism was reported in animal study^30^ and clinical use,^31^ but seldom about IT. In a previous study, we showed that UC‐MSCs, but not AF‐MSCs, may be lethal without cell aggregate removal.^32^ The local administration of IT, enhancing the number of cells that reach the target site. The prevention approach is also clinically relevant as one can predict which premature infants are at high risk for developing BPD. Our results indicated that AF‐, UC‐ and BM‐MSC IT for BPD treatment were safe, consistent with the lack of deaths during the experiments.

MSCs are important modulators of repair after injury, and the biological foundation for MSC cytotherapy is the process of them participation. Pericytes are normally inactive during normal state of the body, and they were activated and considered functional for trophic and immunomodulatory after injury as a kind of MSCs^33^. The transplantation of cultured MSCs was used for medicinal purposes to regenerate the microenvironment during high levels of inflammatory cytokines.^34^ MSCs inhibit immunoactivation‐induced cytotoxicity^34^ and promote endothelial cells forming functional structures.^35^ The mechanism of MSC cytotherapy was to reduce inflammation, prompt angiogenesis and avoid cell death, as it is difficult to be stripped for single factor in vivo study, while those bio‐functions have to be proved by independent experiments in vitro and observed in vivo.^10^ For the hyperoxia‐induced BPD animal model, as shown in a clinical setting, the usage of IT MSC treatment is an effective solution to attenuate its pathological changes,^10^ while the engrafted MSCs share a less than ten percentage of lung after 14 and 21 days after transplanting and adopt a type II alveolar epithelial cell (AEC2) phenotype.^18^ As safety and effectiveness of MSCs treat BPD were taken by some independent study group, and MSCs were already translated in to clinical use for curing BPDs.^4^ In this study, the effectiveness of IT MSC treatments were confirmed by the improved BPD‐associated physiology and biochemical changes in all three MSC treatment groups, but there were differences in groups that need to elucidate clearly before clinical trials.

MSCs from different source present different biological functions and therapeutic effects. As MSCs may be derived from various biological sampling, the difference of them was cared by some independent research groups. The most widely used MSCs were BM‐MSCs and UC‐MSCs, which is derived from Wharton's jelly. The difference between the MSCs involved the WNT pathway growth and differentiation potential.^36^ In the comparison of BM‐MSCs and UC‐MSCs, there were differences in mRNA expression and protein secretion level^37^, ^38^ and UC‐MSCs had enhanced proliferation and immunosuppression ability,^37^, ^39^ which had high expression of IL6, IL8, PDGF, HGF and TGF‐β2.^37^ AF‐MSCs were a mixture of cells derived from lung, kidney and foetal membrane, which is considered as potential for regenerative medicine^40^ and immunomodulatory.^41^ In our previous study, AF‐MSCs were derived from second‐trimester amniotic fluid for cytotoxicity^42^ and cytotherapy^11^, ^32^ study. In vitro, AF‐MSCs and UC‐MSCs shared same CD markers as positive of CD73, CD90, CD147, CD44 and CD29, and negative of CD34, CD14 and CD45, AF‐MSCs got higher mRNA expression level of pluripotency marker (SOX2, NANOG and OCT4) and enhanced differentiation capability of osteogenic and chrongrogenic compared with UC‐MSCs, and there is no difference in the immunosuppression effect of inhibition of PHA‐activated PBMC division.^11^ In vivo, the therapeutic effects of AF‐MSCs and UC‐MSCs in caecal ligation and puncture (CLP)–induced sepsis have been assessed.^32^ AF‐MSCs had less dramatic therapeutic effects compared with UC‐MSCs in lung and kidney injury, but UC‐MSCs had a stronger immunosuppressive effect in lung during sepsis.^32^ Herein, we demonstrated that the three MSCs had therapeutic effects in a hyperoxia‐induced BPD model and that antenatal MSCs (AF‐MSCs and UC‐MSCs) were better at reducing the alveolar dilatation and neovascularization compared with the BM‐MSC group. UC‐MSCs had a better immunosuppressive effect performance compared with AF‐MSCs and BM‐MSCs.

## 5 CONCLUSION

In summary, we confirmed the effectiveness and safety of MSC cytotherapy on a hyperoxia‐induced BPD model and demonstrated that UC‐MSCs were the first choice of BPD treatment in comparison with AF‐MSCs and BM‐MSCs, because UC‐MSCs IT treatment had better therapeutic effects by immunosuppression and promotion of angiogenesis. Furthermore, AF‐MSCs IT treatment was another excellent alternative treatment by autoplastic transplantation. To enhance the clinical therapeutic effects, additional studies are needed to determine the difference of MSCs from different sources.

## CONFLICT OF INTEREST

The authors declare no conflict of interest.

## AUTHOR CONTRIBUTIONS

**Yingjun Xie:** Writing‐original draft (equal). **Fei Chen:** Resources (equal). **Lei Jia:** Data curation (equal). **Rui Chen:** Data curation (equal). **Wei Victor Zhang:** Writing‐review & editing (equal). **XInqi Zhong:** Conceptualization (equal). **Ding Wang:** Data curation (equal); Methodology (equal); Writing‐original draft (equal); Writing‐review & editing (equal).

## Supporting information

Fig S1

## Data Availability

All data generated or analysed during this study are included in this published article.
