# Supplementary figures and images for: Mesenchymal stem cells from different sources show distinct therapeutic effects in hyperoxia‐induced bronchopulmonary dysplasia in rats
Source: J Cell Mol Med. 2021 Jul 29;25(17):8558–66. doi: 10.1111/jcmm.16817 (PMC8419191; doi:10.1111/jcmm.16817)

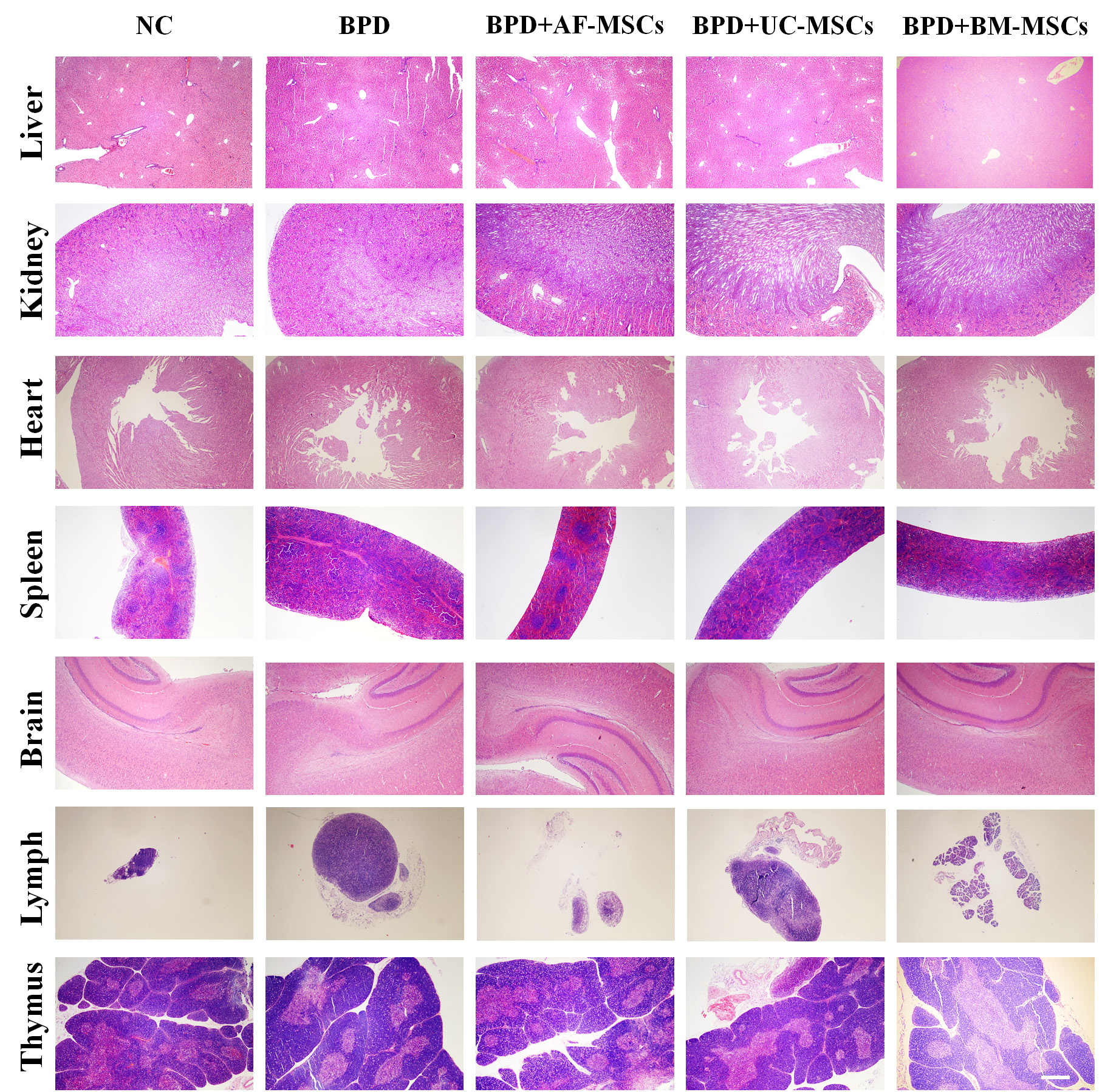

Supplement: Supplementary file 1 — Fig S1 [file JCMM-25-8558-s001.jpg]
